# Effect of high-concentrate diets with calcium lignosulfonate and cottonseed processing method on quantitative traits and non-carcass components of feedlot cull ewes

**DOI:** 10.5194/aab-64-355-2021

**Published:** 2021-09-02

**Authors:** Pablo Teixeira Viana, Gleidson Giordano Pinto de Carvalho, Mirelle Costa Pignata Viana, Dallyson Yehudi Coura de Assis, Mauro Pereira de Figueiredo, Luís Gabriel Alves Cirne, Jennifer Souza Figueredo, Lorena Santos Sousa, Hermógenes Almeida de Santana Júnior, Douglas dos Santos Pina, Henry Daniel Ruiz Alba

**Affiliations:** 1 Universidade Estadual do Sudoeste da Bahia, Itapetinga, Bahia, Code 45700-000, Brazil; 2 Department of Animal Science, Universidade Federal da Bahia, Salvador, Bahia, Code 40170-110, Brazil; 3 Universidade Estadual do Sudoeste da Bahia, Vitória da Conquista, Bahia, Code 45029-066, Brazil; 4 Institute of Biodiversity and Forestry, Universidade Federal do Oeste do Pará, Bahia, Code 68035-110, Brazil; 5 Department of Animal Science, Universidade Federal do Piauí, Corrente, Bahia, Code 64980-000, Brazil

## Abstract

This study examined the effects of cottonseed processing form and the inclusion of calcium lignosulfonate in high-concentrate diets for feedlot cull ewes on carcass traits and non-carcass components. Thirty Santa Inês cull ewes with an average body weight of 44.2 ± 5.2 kg and an average age of 50 months were distributed into collective stalls in a completely randomized design. The treatments consisted of diets including whole cottonseed, crushed cottonseed, whole cottonseed treated with lignosulfonate (100 g/kg, as fed), crushed cottonseed treated with lignosulfonate (100 g/kg, as fed), and a control diet without cottonseed. The experimental diets did not influence (P>0.05) average daily weight gain (0.195 kg/day), slaughter weight (51.74 kg), or in vivo biometric and on-carcass measurements. There was no difference (P>0.05) in loin-eye area or subcutaneous fat thickness as evaluated in vivo by ultrasound. There was no diet effect on hot carcass weight and yield (24.8 kg and 47.8 %), cold carcass weight and yield (24.2 kg and 46.8 %), or chilling loss (2.1 %). Non-carcass components did not differ in response to the diets (P>0.05). Dietary inclusion of calcium lignosulfonate increases the proportions of udder and liver relative to empty body weight (P<0.05). Neither the cottonseed processing method nor the inclusion of calcium lignosulfonate in high-concentrate diets for cull ewes affects their performance, biometric or morphometric measurements, non-carcass components, or qualitative traits of their carcass.

## 1 Introduction

A large portion of the sheep meat consumed in Brazil originates from adult animals, which are mostly culled. However, labels on these products on the market do not provide information on sex, category, age (young or adult), or whether the animal was culled (Pinheiro et al., 2007, 2009). The use of cull animals has been a commonly adopted practice on farms that undertake the entire production cycle, as the sale of these animals is compromised by the low acceptability of their meat (Pelegrini et al., 2008). Furthermore, they do not have the productive and reproductive potential expected of them (Atti et al., 2001).

To increase the profitability of the activity, producers must plan a proper destination for these animals. This is particularly important for females, whose productive and reproductive potential is no longer satisfactory at an earlier age. In this respect, production can be maximized with the adoption of feedlotting, a technique that allows rapid weight gains and, as a consequence, better carcasses.

In recent years, greater value has been placed on edible organs such as offal, skin, and waste used by industries. This, coupled with the association made between these components and carcass yield (that in feedlot sheep heavier weights translate into lower carcass yields), has driven research into non-carcass components (Kuss et al., 2007; Bhatt et al., 2012, 2013; Ben Abdelmalek et al., 2019).

Feed is known to be the costliest factor in an animal production system. In an attempt to lower these costs, by-products emerged as an alternative to replace the most commonly used diet ingredients (corn and soybean meal) without affecting animal productivity or meat quality (da Silva Magalhães et al., 2020; Nascimento et al., 2021; Silva et al., 2021). In this context, oilseeds are lipid sources used to increase the energy density of the diet and improve animal performance (Manso et al., 2006; Homem Júnior et al., 2010) as well as the quality of the carcass and meat (Jerónimo et al., 2009; Atti et al., 2013).

Among the available high-fat options, cottonseed (*Gossypium hirsutum*) – one of the main by-products of the textile industry – should be considered as a protein and energy source for the diet of feedlot-finished cull ewes. However, the limitation of the use of cotton by-products is the presence of gossypol. Gossypol is a polyphenolic compound found in free or bound form (to proteins) throughout the cotton plant. In dairy goats, feeding 350 to 400 mg gossypol/day for 3 months or diets containing ∼ 400 ppm free gossypol are reported to result in toxicosis (Gupta, 2018). In addition, doses ≥ 15 mg/kg body weight of gossypol per day negatively affected the growth of goats (EFSA CONTAM Panel, 2017). On the other hand, it was observed that ruminal doses of 100 µg/mL of gossypol strongly inhibit the growth of all gram-positive bacteria (Wang et al., 2018). Although gossypol is metabolized in the rumen, care must be taken with the amount of this compound in the diet or with the exposure time in the animals (Gupta, 2018).

Calcium lignosulfonate, a by-product of the cellulose industry (Khitrin et al., 2012), is a highly hygroscopic energetic binder that surrounds fatty acids and inhibits bacterial activity. In doing so, it prevents the deleterious effects of fat and protects grain nutrients from rumen degradation, thereby reducing the availability of lipids in the rumen (Neves et al., 2009) and maximizing intestinal absorption.

In view of the above-described facts, the present study was developed to investigate the effects of cottonseed in different processing forms and the inclusion of calcium lignosulfonate in high-concentrate diets for feedlot cull ewes on carcass traits and non-carcass components.

## 2 Materials and methods

### 2.1 Location and experimental period

The experiment was conducted on Malhada das Cabaças Farm (14${}^{\circ }$13${}^{\prime }$ S, 42${}^{\circ }$46${}^{\prime }$51${}^{\prime \prime }$ W; 525 a.s.l.), where the average annual precipitation is 553.5 mm, and at the Guanambi slaughterhouse (FGI). Both locations are situated in Guanambi – BA, Brazil. All animal procedures were previously approved by the Ethics Committee at the State University of Southwestern Bahia, Brazil (approval no. 042/2013).

### 2.2 Animals, experimental design and diets

Thirty Santa Inês cull ewes with an average initial live weight of 44.2 ± 5.2 kg and an average age of 50 months were distributed in a completely randomized design. Sanitary measures against endo- and ectoparasites were taken and the ewes were housed in collective sawdust-bedded stalls (2.0 × 5.0 m) at the rate of six animals per stall. The stalls were equipped with collective polyethylene feeders (0.5 m/animal) and drinkers, which were cleaned daily.

Five high-concentrate experimental diets were prepared. The diets were formulated with 100 % concentrate, without roughage. The feed was supplied twice daily (half at 07:00 and the other half at 15:00) and adjusted daily to allow orts around 15 % of the total supplied, characterizing *ad libitum* intake. The diets were formulated as recommended by NRC (2007) and supplied so as to meet the daily requirements for an average daily gain of 300 g. Treatments were represented by the physical form of cottonseed (whole or crushed) and the inclusion of calcium lignosulfonate (Table 1) in the diet as follows: WCS – whole cottonseed; CCS – crushed cottonseed; WCSL – whole cottonseed with lignosulfonate (100 g/kg of cottonseed as fed); CCSL – crushed cottonseed with lignosulfonate (100 g/kg of cottonseed as fed); and CON – control diet, without cottonseed.

**Table 1:** Proportion of ingredients and physical and chemical composition of experimental diets.

Item	Diet${}^{a}$				
	CON	WCS	CCS	WCSL	CCSL
Ingredients (g/kg dry matter)					
Corn	680	580	580	580	580
Soybean meal	190	40	40	40	40
Cottonseed	–	340	340	340	340
Cottonseed cake	100	–	–	–	–
Urea	–	10	10	10	10
Vitamin–mineral premix${}^{b}$	30	30	30	30	30
Chemical composition (g/kg dry matter)					
Dry matter (g/kg as fed)	897.5	881.2	895.3	894.6	887.9
Organic matter	96.35	96.74	96.69	95.98	95.81
Crude protein	180.4	182.6	168.8	171.5	168.0
Ether extract	27.7	76.6	71.5	78.0	66.9
Neutral detergent fiber	232.8	310.1	305.9	313.8	314.6
Neutral detergent fiber${}_{\mathrm{ap}}^{c}$	175.5	273.2	263.2	276.2	272.9
Acid detergent fiber	108.0	157.7	141.8	134.6	154.6
Indigestible acid detergent fiber	34.5	72.1	60.2	78.9	59.4
Cellulose	86.3	104.3	89.7	86.6	112.1
Hemicellulose	124.8	152.5	164.0	179.0	160.0
Lignin	21.6	53.4	52.1	48.0	42.5
Mineral matter	47.5	42.4	45.9	43.2	49.2

${}^{a}$ CON = control diet; WCS = whole cottonseed; CCS = crushed cottonseed; WCSL = whole cottonseed treated with calcium lignosulfonate (100 g/kg); and CCSL = crushed cottonseed treated with calcium lignosulfonate (100 g/kg). ${}^{b}$ Composition: saline purgative UCB^®^ – 16.7 %; ADE vitamin powder – 16.7 %; and mineral supplement – 66.7 %. ${}^{c}$ Corrected for ash and protein.

The experiment lasted 54 d, of which the first 12 were used for the animals to adapt to the collective stalls and dietary management. In the diets, the concentrate was added replacing hay of *Digitaria decumbens * Stent. cv. Transvala gradually, every 4 d, at the following ratios: 60:40 (day 1); 80:20 (day 5); and 100:0 (day 9). The first evaluation took place on the 13th day, when the total diet had 100 % concentrate, and the evaluation period was 42 d, of which the last 5 d were used for data collection.

### 2.3 Slaughter and carcass traits

Prior to slaughter, the ewes were weighed to determine slaughter weight (SW) and calculate the average daily weight gain (ADG).

Then, in vivo measurements of subcutaneous fat thickness (SFT) and loin-eye area (LEA) were performed using an ultrasound device (ALOKA^®^ SSD 500 v) with a 3.5 MHz linear transducer, a 12 cm acoustic probe, and a silicone coupler. After images were captured, the ewes were shorn and clipped in the region between the 12th and 13th thoracic vertebrae, on the left side. The best image of the loin-eye area was captured by measuring it from the medial side of the *Longissimus* muscle to its lateral side of the midline. The length (A, cm) and maximum depth (B, cm) of the muscle were measured to calculate LEA (in cm${}^{2}$) using the following ellipse formula: LEA = A/2 × B/2 × π (Silva Sobrinho et al., 2003). Using the same image, SFT was also determined (in mm) over the said muscle area.

Next, the following in vivo biometric measurements were taken with the animals standing on a flat surface: rump height, withers height, body length, chest width, chest girth, rump width, shank circumference, leg length, and shank length. Length and circumferences measurements were taken using a tape measure, whereas the width and depth variables were measured with a measuring stick. Body compactness was determined as the ratio between slaughter weight and body length.

At the time of slaughter, the animals were transferred to the slaughterhouse located in Guanambi – BA, Brazil, where they were slaughtered under veterinary inspection following the current norms established by the Regulation for Industrial and Sanitary Inspection of Products (Brazil, 2000).

After bleeding and skinning, the carcasses were eviscerated and the non-carcass components were separated into rumen–reticulum, omasum–abomasum, small and large intestines, heart, liver, kidneys, lungs–trachea–esophagus, tongue, blood, and external body components (head, feet, and skin). The gastrointestinal tract (GIT) was initially weighed full. Then it was emptied, washed and weighed again to determine the GIT content and calculate empty body weight (EBW = SW – GIT content). Non-carcass components were estimated relative to EBW.

Subsequently, the hot carcass weight (HCW) was determined and the hot carcass yield (HCY) was calculated by the following equation: HCY = HCW/SW × 100 (Cézar and Souza, 2007).

After 24 h of refrigeration in a cold room at 4 ${}^{\circ }$C, the carcasses were weighed to determine cold carcass weight (CCW) and assessed subjectively for conformation [1 = Poor (concave); 2 = Regular (sub-concave); 3 = Good (straight); 4 = Very good (sub-convex); 5 = Excellent (convex)] and degree of fatness (1 = Emaciated; 2 =  Lean; 3 = Medium; 4 = Fat; 5 = Obese). The pelvic–renal fat, or visceral fat, was assessed by assigning scores from 1 to 3, where 1 = Low (both kidneys uncovered); 2 = Normal (one kidney covered); and 3 = Excessive (both kidneys covered).

Following the subjective assessment of pelvic–renal fat, the kidneys and pelvic–renal fat were removed and their weights were recorded and subtracted from the hot and cold carcass weights. Next, the cold carcass yield (CCY) and chilling loss (CL) were calculated using the following formulas: CCY = CCW/SW × 100 and CL = (HCW – CCW) / HCW × 100.

Whole carcass length, internal carcass length, shank circumference, shank length, chest depth, chest width, chest girth, chest circumference, rump circumference, and rump width were measured. Carcass capacity was determined as the ratio between cold carcass weight and internal carcass length.

After the morphological evaluation, the carcasses were sectioned lengthwise, along the spine with an electric saw, which resulted in the separation of the right and left half-carcass. The left half-carcass was used for direct (on-carcass) measurements of LEA and SFT. Loin-eye area was determined by making a cross section between the 12th and 13th thoracic vertebrae and tracing the outline of the muscle on acetate sheet, in correspondence with the cranial portion of the loin. The maximum distance and depth of the muscle were measured with a ruler and calculated from the formula proposed by Silva Sobrinho et al. (2003), as previously mentioned in the ultrasound evaluation of LEA. The subcutaneous fat thickness in the carcass was measured (in mm) using a digital caliper at a 3/4 distance from the medial side of the *Longissimus* muscle, towards the side of the spinal process.

### 2.4 Statistical analyses

The effects of dietary treatments on the measured parameters were analyzed according to the following model:

$${\hat{Y}}_{ij}=\mu +D_{i}+\varepsilon_{ij},$$

where ${\hat{Y}}_{ij}$ is the measurement j of diet i, μ is the overall mean, $D_{i}$ is the fixed effect of diet i (i = C, WCS, CCS or CCSL), and $\varepsilon_{ij}$ is the random error term.

Additionally, the following contrasts were used to compare the effects of the different diets:

- Contrast 1: cottonseed effect {CON vs. (WCS + CCS + WCSL + CCSL)}
- Contrast 2: lignosulfonate effect {(WCS + CCS) vs. (WCSL + CCSL)}
- Contrast 3: cottonseed physical form effect {(WCS + WCSL) vs. (CCS + CCSL).}

All statistical procedures were performed using the PROC GLM procedure of the SAS software (SAS, 2014), adopting 0.05 as the critical probability of type-I error.

## 3 Results

### 3.1 Performance and biometric measurements

Neither the cottonseed processing method nor the inclusion of calcium lignosulfonate compromised the ADG or SW of the feedlot ewes. The control diet in comparison to the others (CON vs. other diets), cottonseed processing method (WCS + WCSL vs. CCS + CCSL), and calcium lignosulfonate inclusion in the high-concentrate diets (WCS + CCS vs. WCSL + CCSL) also did not influence (P>0.05) ADG (0.195 g) or SW (51.74 kg) (Table 2).

**Table 2:** Weight gain, biometric measurements, subcutaneous fat thickness (SFT), and loin-eye area (LEA) of the *longissimus* muscle and body capacity obtained in vivo by ultrasound in Santa Inês cull ewes fed high-concentrate diets with cottonseed associated with calcium lignosulfonate.

Item	Diet${}^{a}$					SEM	Contrast${}^{b}$		
	CON	WCS	CCS	WCSL	CCSL		1	2	3
Initial live weight (kg)	46.0	43.5	44.7	42.5	44.5	2.279	0.8145	0.9107	0.5711
Average daily gain (kg)	0.209	0.165	0.192	0.223	0.186	0.023	0.8780	0.0960	0.5022
Slaughter weight (kg)	54.9	49.4	50.8	51.8	51.8	0.454	0.7000	0.3233	0.8881
Rump height (cm)	74.4	71.2	70.5	71.2	74.0	0.695	0.2131	0.8969	0.8969
Withers height (cm)	75.4	72.5	71.7	72.5	74.0	0.692	0.2667	0.6496	0.6496
Body length (cm)	49.4	48.1	46.5	48.6	49.0	1.174	0.8780	0.0960	0.9672
Chest width (cm)	19.1	19.1	18.3	19.4	18.7	0.322	0.3715	0.6504	0.9536
Chest depth (cm)	28.9	29.0	29.2	28.1	27.6	0.433	0.4777	0.8352	0.2802
Chest circumference (cm)	90.2	87.7	89.0	89.0	89.2	0.726	0.9963	0.4647	0.9258
Rump width (cm)	19.0	18.9	18.2	20.3	19.1	0.437	0.0507	0.1579	0.2352
Shank circumference (cm)	37.6	36.5	35.8	37.5	35.0	0.836	0.6945	0.3369	0.6672
Leg length (cm)	44.2	43.7	42.7	42.7	44.8	0.676	0.4862	0.5877	0.9029
Shank length (cm)	20.2	17.5	17.8	18.5	19.2	0.272	0.1434	0.1010	0.9780
Body compactness (kg/cm)	1.10	1.03	1.13	1.06	1.06	0.020	0.1461	0.2742	0.7125
LEAU (cm${}^{2})$	8.7	7.0	7.3	8.5	8.4	0.240	0.1684	0.1236	0.2866
SFTU (mm)	1.8	1.5	1.7	1.8	1.7	0.090	0.8807	0.2465	0.6155
MUCC (%)	64.3	68.8	64.2	65.5	62.6	1.802	0.8190	0.8352	0.5421
MULW (%)	16.2	14.3	14.5	16.5	16.2	0.486	0.2807	0.3327	0.3108

${}^{a}$ CON = control diet; WCS = whole cottonseed; CCS = crushed cottonseed; WCSL = whole cottonseed treated with calcium lignosulfonate (100 g/kg); and CCSL = crushed cottonseed treated with calcium lignosulfonate (100 g/kg). LEAU = loin-eye area measured by ultrasonography; SFTU = subcutaneous fat thickness measured by ultrasonography; MUCC (LEAU/CCW) = muscularity measured by ultrasonography relative to cold carcass weight; and MULW ((LEAU/FLW) × 100) = muscularity measured by ultrasonography relative to final live weight. SEM = standard error of the mean. ${}^{b}$ 1 = CON vs. other treatments; 2 = (WCS + CCS) vs. (WCSL + CCSL); and 3 = (WCS + WCSL) vs. (CCS + CCSL).

There were no differences (P>0.05) in body compactness (1.07 kg/cm), LEA (8.0 cm${}^{2})$, or SFT (1.70 mm) evaluated in vivo by ultrasound or in muscularity relative to carcass weight and final live weight (Table 2).

In comparison with the control diet without cottonseed, the inclusion of calcium lignosulfonate (WCS + CCS vs. WCSL + CCSL) and the cottonseed processing form (WCS + WCSL vs. CCS + CCSL) did not affect (P>0.05) the quantitative traits of HCW and HCY (24.8 kg and 47.8 %), CCW and CCY (24.2 kg and 46.8 %), or chilling loss (2.1 %) (Table 3). The treatments also did not affect (P>0.05) the subjective measurements of conformation (1–5), degree of fatness (1–5), or visceral fat (1–3) evaluated on the carcass, which averaged 2.6, 3.10, and 2.6 points, respectively (Table 3). There was also no effect of calcium lignosulfonate inclusion or the cottonseed processing method on LEA or SFT measured on the carcass, whose mean values were found to be 15.6 cm${}^{2}$ and 0.88 mm, respectively (Table 3).

### 3.2 Morphometric measurements and non-carcass components

The morphometric measurements evaluated on the carcass did not differ (P>0.05) between the treatment groups. The mean values of the evaluated variables were as follows: total carcass length: 56.8 cm; internal carcass length: 65.9 cm; shank length: 44.1 cm; chest depth: 26.1 cm; chest width: 29.6 cm; chest girth: 19.2 cm; rump circumference: 58.0 cm; and rump width: 19.1 cm (Table 4).

In the analysis of non-carcass components, neither calcium lignosulfonate inclusion nor the cottonseed processing method influenced (P>0.05) the full or empty GIT, rumen–reticulum, omasum–abomasum and empty intestines, or inguinal, pelvic, omental, renal, and total fats. However, calcium lignosulfonate inclusion in the diet increased the proportions of udder (P=0.0453) and liver (P=0.0458) relative to EBW (Table 5).

## 4 Discussion

### 4.1 Performance and biometric measurements

The ADG and SW of the feedlot ewes evaluated in this study were considerably higher than the 0.034 and 45.21 kg, respectively, reported by Souza et al. (2010) in Barriga Preta feedlot ewes fed high-concentrate diets, demonstrating the higher potential of Santa Inês ewes for performance and meat production.

Rump and withers heights were similar (P>0.05) between the treatments, averaging 72.3 and 73.2 cm, respectively (Table 4). This indicates that the animals had a uniform body height, which is a very important trait to accurately determine other biometric measurements in vivo and on the carcass (Table 5) (Pinheiro and Jorge, 2010; Pinheiro et al., 2015). Supporting these assumptions, the results of our study were practically identical to those observed by Pinheiro and Jorge (2009), who found 69.5 and 68.0 cm for rump and withers heights, respectively, in Santa Inês sheep with the same SW (43 kg).

The morphometric measurements of the cull ewes did not differ between the treatments (P>0.05). As the tested diets did not influence these measurements, it is inferred that there was a common body condition pattern, considering that a selection process was performed to acquire animals at approximate ages, which was achieved by evaluating their dental arch. According to Garcia et al. (2003), another explanation is that cull animals are mostly at a stage when their physiological growth has already concluded and their body condition will be a determining factor for their biometrics. Yet another factor that may explain the similar results is that ADG and SW were not affected. This supports the affirmation of Marques et al. (2008), who asserted that biometric measurements are little influenced by nutritional management provided that the animals are slaughtered with the same weight.

**Table 3:** Performance and qualitative traits of the carcass of Santa Inês cull ewes fed high-concentrate diets with cottonseed associated with calcium lignosulfonate.

Item	Diet${}^{a}$					SEM	Contrast${}^{b}$		
	CON	WCS	CCS	WCSL	CCSL		1	2	3
Hot carcass weight (kg)	26.5	23.0	24.9	24.8	24.6	1.226	0.9036	0.1988	0.9624
Cold carcass weight (kg)	25.9	22.5	24.3	24.3	24.0	0.637	0.9260	0.1986	0.9805
Hot carcass yield (%)	48.4	46.6	48.9	47.8	47.5	0.613	0.2388	0.3256	0.8528
Cold carcass yield (%)	47.2	45.6	47.8	46.8	46.5	0.465	0.2795	0.3629	0.8469
Chilling loss (%)	2.3	2.0	2.3	2.0	2.1	0.465	0.4772	0.6518	0.7405
Conformation (1–5)	2.9	3.0	3.1	3.2	3.0	0.085	0.7008	0.7744	0.6334
Degree of fatness (1–5)	2.7	3.2	3.3	3.2	3.0	0.110	0.3929	0.4126	0.5377
Visceral fat (1–3)	2.8	2.5	2.5	2.7	2.3	0.108	0.7800	0.1946	0.5335
LEA (cm${}^{2})$	16.3	15.5	15.4	15.6	15.1	0.378	0.8521	0.3967	0.5016
SFT (mm)	0.70	0.95	1.00	1.08	0.71	0.088	0.5307	0.7655	0.7332
Left loin (kg)	0.243	0.272	0.254	0.262	0.256	0.006	0.7797	0.4126	0.9022

${}^{a}$ CON = control diet; WCS = whole cottonseed; CCS = crushed cottonseed; WCSL = whole cottonseed treated with calcium lignosulfonate (100 g/kg); and CCSL = crushed cottonseed treated with calcium lignosulfonate (100 g/kg). SEM = standard error of the mean. ${}^{b}$ 1 = CON vs. other treatments; 2 = (WCS + CCS) vs. (WCSL + CCSL); 3 = (WCS + WCSL) vs. (CCS + CCSL). LEA = loin-eye area measured on the carcass; SFT = subcutaneous fat thickness measured on the carcass.

**Table 4:** Morphometric measurements and quantitative traits of the carcass of Santa Inês cull ewes fed high-concentrate diets with cottonseed associated with calcium lignosulfonate.

Item	Diet${}^{a}$					SEM	Contrast${}^{b}$		
	CON	WCS	CCS	WCSL	CCSL		1	2	3
Total carcass length (cm)	56.6	56.5	57.8	55.5	57.7	0.778	0.5136	0.5510	0.9846
Internal carcass length (cm)	68.4	65.0	63.7	65.3	67.0	1.050	0.2935	0.7103	0.8190
Shank circumference (cm)	45.2	44.1	43.1	42.8	43.1	1.000	0.5586	0.7479	0.5222
Shank length (cm)	42.6	43.5	42.5	41.8	43.5	1.554	0.7928	0.2978	0.7534
Chest depth (cm)	26.6	26.3	26.0	25.8	25.8	0.352	0.8640	0.8649	0.4218
Chest width (cm)	30.4	29.5	30.6	27.3	30.4	0.539	0.3690	0.3732	0.3732
Chest girth (cm)	19.8	18.9	19.6	18.4	19.5	0.314	0.5690	0.8579	0.4981
Chest circumference (cm)	86.8	81.3	84.5	81.2	82.5	2.303	0.5570	0.3861	0.1519
Rump circumference (cm)	60.0	54.0	59.5	57.5	58.8	1.143	0.5015	0.3622	0.6466
Rump width (cm)	18.7	19.3	19.1	19.3	19.1	0.282	0.9620	0.7296	0.7296
Carcass capacity (kg/cm)	0.379	0.347	0.395	0.371	0.360	0.012	0.1460	0.2741	0.5342

${}^{a}$ CON = control diet; WCS = whole cottonseed; CCS = crushed cottonseed; WCSL = whole cottonseed treated with calcium lignosulfonate (100 g/kg); and CCSL = crushed cottonseed treated with calcium lignosulfonate (100 g/kg). SEM = standard error of the mean. ${}^{b}$ 1 = CON vs. other treatments; 2 = (WCS + CCS) vs. (WCSL + CCSL); 3 = (WCS + WCSL) vs. (CCS + CCSL).

The average body compactness indices found in the ewes of the present study were higher than the 0.61 described by Pinheiro and Jorge (2010). This may have been due to the better nutritional composition of the diet supplied in this study (17.38 vs. 11.81 % CP), which resulted in higher weight gain (SW = 51.74 vs. 43.34 kg; Pinheiro et al., 2015) and, consequently, greater muscle deposition in the carcass (LEA = 15.58 vs. 11.92 cm${}^{2}$; Pinheiro et al., 2009). Body compactness is an important parameter, as it can influence the product's selling price and its visual perception. In addition, it favors the consumption of this meat, since higher body compactness indices translate into higher proportions of muscles and fat in the animal (Costa Júnior et al., 2006).

**Table 5:** Non-carcass components (% of the empty body weight) of Santa Inês cull ewes fed high-concentrate diets with cottonseed associated with calcium lignosulfonate.

Item	Diet${}^{a}$					SEM	Contrast${}^{b}$		
	CON	WCS	CCS	WCSL	CCSL		1	2	3
Empty body weight (kg)	51.5	46.1	47.8	48.5	48.8	1.054	0.7357	0.3313	0.9564
Head	4.457	4.595	4.710	4.611	4.569	0.046	0.2028	0.6505	0.5449
Blood	3.783	3.894	3.546	3.689	3.711	0.081	0.3022	0.7281	0.4706
Feet	1.872	1.874	1.796	1.856	1.996	0.049	0.4289	0.5442	0.6494
Skin	9.123	8.124	8.640	9.511	7.617	0.205	0.9162	0.1001	0.8796
Reproductive tract	0.689	0.860	1.272	0.719	0.760	0.099	0.4444	0.6299	0.8725
Udder	0.671	0.452	0.627	0.656	0.548	0.036	0.6067	0.0453	0.6069
Heart	0.874	0.773	0.819	0.812	0.861	0.019	0.8115	0.5557	0.7596
Liver	2.176	2.018	1.937	2.195	1.866	0.054	0.3400	0.0458	0.5704
Respiratory tract	2.212	2.261	2.052	2.159	1.983	0.067	0.5610	0.6855	0.2954
Kidneys	0.312	0.251	0.265	0.324	0.308	0.014	0.3358	0.2156	0.2702
Tongue + esophagus	0.379	0.479	0.430	0.425	0.437	0.012	0.9929	0.3049	0.9368
Rumen–reticulum	2.540	2.796	2.555	2.659	2.449	0.058	0.7006	0.8601	0.3880
Omasum–abomasum	0.889	0.976	0.916	0.863	0.871	0.039	0.8776	0.6131	0.4805
Intestine	3.267	3.459	2.855	3.338	2.886	0.149	0.3261	0.7063	0.4699
Gastrointestinal tract content	1.627	1.986	1.783	1.730	1.919	0.457	0.7591	0.7345	0.8493
Renal fat	2.201	1.792	2.082	2.253	2.015	0.089	0.9419	0.1224	0.5023
Inguinal fat	1.484	1.394	1.276	1.382	1.622	0.066	0.2557	0.6192	0.6784
Pelvic fat	0.499	0.488	0.422	0.552	0.418	0.021	0.1973	0.1221	0.8616
Omentum	6.822	6.623	6.309	6.313	6.143	0.224	0.7808	0.7294	0.3584
Total fat	1.101	1.030	1.009	1.050	1.020	0.274	0.5726	0.4381	0.6428

${}^{a}$ CON = control diet; WCS = whole cottonseed; CCS = crushed cottonseed; WCSL = whole cottonseed treated with calcium lignosulfonate (100 g/kg); and CCSL = crushed cottonseed treated with calcium lignosulfonate (100 g/kg). SEM = standard error of the mean. ${}^{b}$ 1 = CON vs. other treatments; 2 = (WCS + CCS) vs. (WCSL + CCSL); 3 = (WCS + WCSL) vs. (CCS + CCSL).

The LEA and SFT results obtained by ultrasound may be attributed to the fact that the animals were slaughtered at similar body weights. This finding corroborates the following inference drawn by Osório (2002): when carcasses have similar weights and amounts of fat, almost all body regions have similar proportions, irrespective of the breed. Loin-eye area and SFT are traits used as indicators of muscularity and fatness, respectively (Souza Júnior et al., 2013; Al-Jammas et al., 2016).

In the current study, HCY and CCY were higher than the 45.0 % and 44.7 % reported by Pinheiro et al. (2009) and 45.2 % and 43.7 % described by Pelegrini et al. (2008) in Santa Inês and ideal feedlot cull ewes, respectively. These results confirm the presuppositions that when fed diets of better nutritional quality and specialized in and having potential for meat production, feedlot ewes exhibit higher performance and, consequently, higher carcass yields.

### 4.2 Morphometric measurements and non-carcass components

The morphometric traits of a carcass vary according to breed, sex, and diet (Murta et al., 2009). Therefore, the similarity found between the treatment groups in this study can be explained by the similar composition of the diets, since the animals shared the same sex, breed, age, and live weight. In addition, growth in adult animals is limited, which results in similar body measurements (Garcia et al., 2003).

According to Pinheiro et al. (2009), in sheep, changes in skin and udder weight are directly related to the animal's age and physiological stage. The liver and renal fat, on the other hand, are more affected by compensatory gain. Thus, part of the weight changes is the result of the recovery of the liver's metabolic activity and, consequently, the increased weight of these organs. This may explain the heavier weight of the udder and liver in the animals that received diets with inclusion of calcium lignosulfonate, which exhibited a higher absolute weight at slaughter (Table 4).

Calcium lignosulfonate improves nitrogen metabolism by increasing recycling (Cirne et al., 2020). This effect probably contributes to increasing liver activity and, as a consequence, animal size. Additionally, this enhancement can increase the number of cells in the udder or its activity by increasing its size.

The cottonseed-containing diets, whose neutral detergent fiber (NDF) content was higher due to the ingredient, were expected to result in a larger GIT content and, consequently, a higher volume of its components, given the longer residence time of this feed in the rumen. Alves et al. (2013) and Medeiros et al. (2008) observed this phenomenon in their experiment, where they describe higher GIT contents in Santa Inês and Morada Nova sheep, respectively, which were fed a high-fiber diet. Nonetheless, the increase in GIT content is thought to be greater when diets rich in roughage-derived fiber are used. Moreover, because the animals received diets with a similar physicochemical composition and were subjected to the same pre-slaughter fasting time, this response may be considered biologically normal.

## 5 Conclusions

Neither the cottonseed processing method nor the inclusion of calcium lignosulfonate in high-concentrate diets affects the performance, biometric or morphometric measurements, non-carcass components, or qualitative traits of the carcass of cull ewes. In addition, the inclusion of calcium lignosulfonate increases the proportions of liver and udder relative to empty body weight.

Further research is warranted to elucidate the effect of cottonseed processing method and the inclusion of calcium lignosulfonate in high-concentrate diets for sheep.

## Data Availability

The original data used in this study are available from the corresponding author upon request.
